# Exploring the biological function of dreaming: lessons learned from recruitment challenges in a pilot study with PCA stroke patients

**DOI:** 10.3389/frsle.2025.1585263

**Published:** 2025-07-21

**Authors:** Tamara Fischmann, Luisa Peters, Vanessa Rieker, Michael Koslowski

**Affiliations:** ^1^Department of Clinical Psychology and Psychoanalysis, International Psychoanalytic University Berlin, Berlin, Germany; ^2^Department of Psychiatry and Psychotherapy, Charité Universitätsmedizin, CCM - Berlin, Berlin, Germany

**Keywords:** dreaming, REM sleep, posterior stroke, PCA stroke, recruitment challenges, non-declarative memory consolidation

## Abstract

**Background:**

This methodological report draws conclusions about a study that aimed to investigate the biological function of dreams. The main hypothesis in the study posited that dreams serve to maintain sleep by hallucinatory wish fulfillment in response to affect-laden impulses. A secondary hypothesis was whether dreams contribute to the consolidation of non-declarative memory. Neurological patients with ischemic stroke of the posterior cerebral artery (PCA stroke) served as a pragmatic lesion model for loss of dreaming ability, following evidence on the importance of parieto-occipital cortical areas for dream formation.

**Methods:**

This quasi-experimental, two-arm controlled study intended to assess sleep quality and non-verbal memory consolidation in participants with PCA stroke who lost dreaming ability, compared to PCA stroke patients with preserved dreaming. Descriptive statistics using morbidity variables (e.g., NIHSS, Barthel-Index, and thrombolysis) were conducted to assess recruitment challenges.

**Results:**

Recruitment was significantly constrained due to inclusion criteria and multimorbidity of the study population. Of 255 patients screened, 14 were included, and 6 completed the study. Descriptive data from the 14 inclusions as well as reasons for exclusion and dropouts are reported. Morbidity variables varied substantially between completers and non-completers, indicating that PCA stroke might not be an appropriate lesion model for dream research.

**Conclusion:**

This methodological report highlights the difficulties in recruiting adequate patient samples for dream research within a stroke lesion model, in order to gain valuable insights into methodological obstacles in this field. Solutions to these challenges are proposed, e.g., using functional lesion models in healthy controls with transcranial alternating current stimulation (tACS).

**Trial registration:**

clinicaltrials.gov (ID: NCT04749992).

## Introduction

This methodological report explores the complex phenomena of dreaming,^1^ aiming to provide a comprehensive understanding of its biological function separate from the state of REM sleep.

In our study, “dreaming” refers to the subjective experience of perceptual, cognitive, and emotional phenomena that occur during sleep. This includes the narrative and emotional qualities of dreams as reported by participants. Our primary objective was to investigate how this dreaming experience—if it occurs—affects sleep architecture and memory consolidation.

Across the history of dream study, considerable attention has been paid to the origins and functions of dreams, beginning with Freud's seminal work, which posited that dreams act as a mechanism for fulfilling unconscious wishes to protect the sleeper from waking prematurely (Freud, 1900). Despite more than a century of exploration, the question of why we dream remains an open and active area of research.

Over the years, a variety of theories have emerged to explain the multifaceted role of dreaming in both biological and psychological contexts. Evolutionary perspectives, such as the threat simulation theory, suggest dreams offer a rehearsal space for threats, thereby providing a survival advantage (Revonsuo, 2000). The memory consolidation hypothesis suggests that dreaming is vital for integrating new information and emotions, ultimately shaping long-term memory (Nir and Tononi, 2010). Meanwhile, the protoconsciousness theory proposes that dreams serve as developmental tools refining and supporting conscious experience (Hobson and McCarley, 1977). These insights are complemented by emerging neuroimaging studies, which reveal the complex interplay of brain networks during sleep, highlighting that no single explanation can fully account for the diverse functions of dreaming (Domhoff, 2017). Additionally, various theories emphasize dreaming's role in emotional regulation and problem-solving (Cartwright, 1974; Barrett, 1993), further underscoring the necessity for interdisciplinary research—encompassing neuroscience, psychology, and evolutionary biology—to comprehensively elucidate the biological functions of dreaming.

In examining the biological and psychological underpinnings of dreams, the activation-synthesis model posited by Hobson and McCarley (1977) suggests that dreams may emerge from random activations in the visual cortex instigated by brainstem neural activity during REM sleep, positioning dreams as potentially meaningless byproducts of these processes. Contrary to this, other perspectives argue that dreaming occurs independently from REM activity, with dream formation influenced by neural activity in dopaminergic forebrain regions (Solms, 2000). Studies of patients with cortical lesions have shown that damage to specific brain regions such as the parieto-occipital area can lead to a loss of dream recall, suggesting varied neural pathways may influence dreaming (Solms, 2014). Further research indicates that thrombotic infarctions in the PCA territory can also result in dream loss despite preserved REM sleep, offering a unique vantage for investigating these phenomena (Bischof and Bassetti, 2004). Following these observations, Solms and colleagues conducted a study comparing patients with and without dreaming capabilities post-PCA infarction, revealing significant differences in sleep behavior, including sleep duration and frequency of awakenings, arousals, and micro-arousals (unpublished data).

It has also been proposed that the appearance of memories in dreams can promote learning by (1) reactivating memory elements in their original perceptual form, (2) strengthening and consolidating these elements through connecting them with other memory traces, and (3) facilitating the later retrieval of newly learned information (Nielsen and Stenstrom, 2005; Payne and Nadel, 2004).

The intended study deliberately focused on neurological patients with posterior cortical lesions, hypothesizing that these individuals, at risk of losing their dreaming capability, will provide distinct insights into the specific function of dreaming, with the aim of disentangling the process of dreaming from its traditionally linked REM state by examining populations who have experienced thrombotic infarctions within the posterior territory of the posterior cerebral artery (PCA)—particularly those who maintain REM sleep but either lose or retain their ability to dream.

By harnessing neuropsychological and neuroimaging evidence, this methodological approach aims to clarify the independence and biological significance of dreams, potentially expanding its implications across sleep medicine, neuroscience, and clinical neurology. This examination addresses whether dreaming influences the affective consolidation of memories and seeks to elucidate the broader impact of dream loss on emotional regulation and memory processing. The methodological framework outlined in this study holds potential for advancing our understanding of dreaming's intricate role in the human cognitive and emotional landscape, with significant ramifications for therapeutic strategies in clinical settings.

## Methods

### Study design

This study was conducted as a prospective, two-arm controlled observational study employing a quasi-experimental between-group design. Participants were assigned to an experimental group (non-dreamers) or a control group (dreamers) based on their ability to recall dreams after a posterior cerebral artery (PCA) infarction, subjectively assessed during screening and later verified by asking for a dream report during REM phase awakenings in the sleep lab.

The experimental group consisted of patients who reported a loss of dreaming post-stroke, while the control group patients retained the ability to recall dreams. Solms' pilot study showed that from 100 recruited patients approximately 30% of patients were non-dreamers. A statistical power analysis was conducted prior to the beginning of our study to determine the sample size. Based on a two-sided *t*-test for independent samples, assuming an expected effect size of d = 0.68., a significance level of α = 0.05, and a power of 0.80, a total sample size of *N* = 70 (35 participants per group) was required. Given an anticipated attrition rate of approximately 60% due to screening exclusions and dropout, a substantially larger number of patients was initially approached for recruitment.

### Participants

Participants were eligible if they met the following inclusion criteria: (1) documented acute thrombotic infarction in the PCA territory, specifically involving parieto-temporo-occipital regions; (2) pre-infarction dream recall frequency exceeding one dream per week; (3) presence of REM sleep; (4) age between 18 and 90 years; (5) willingness to participate in sleep laboratory examinations as part of the study and to complete questionnaires/diaries; (6) cognitive and linguistic capacity to comprehend study procedures; and (7) willingness to participate, as evidenced by written informed consent.

The exclusion criteria were: (1) extension of the infarction to the brainstem; (2) sleep disorders confounding results, such as severe insomnia, obstructive sleep apnea (OSA), restless leg syndrome (RLS), or periodic limb movements during sleep (PLMS); (3) neurological or psychiatric disorders influencing sleep; (4) cerebral infarctions outside the PCA territory (excluding small vessel disease); and (5) use of medications affecting sleep architecture (e.g., benzodiazepines, anticonvulsants, selective serotonin reuptake inhibitors).

### Recruitment and study procedure

Patients were recruited from the “1,000 Plus” and “LOBI” databases (for details see NCT00715533 and NCT02077582 on clinicaltrials.org), as well as from ongoing patient care in three neurological stroke units at Charité - Universitätsmedizin Berlin, in collaboration with the Center for Stroke Research Berlin (CSB). Eligible patients with PCA infarctions were identified and contacted for telephone screening, which involved assessments of pre- and post-stroke dream activity and evaluation of exclusion criteria using standardized questionnaires: The Insomnia Severity Index (ISI; cutoff ≥15; Morin, 1993), the Restless Leg Syndrome Diagnostic Index (RLS-DI; cutoff ≥11; Benes et al., 2005), and the STOP-BANG questionnaire for obstructive sleep apnea (OSA**;** cutoff ≥5; Chung et al., 2016).

Ethics approval was obtained (May 14, 2019) via the Ethics Board at Campus Charité – Mitte (EA1/111/19). The study protocol was pre-registered at clinicaltrials.gov (ID: NCT04749992).

Visit 1: Participants were provided with detailed verbal and written explanations of the study protocol. Written informed consent was obtained. Sociodemographic and clinical data were collected, including assessments using the Mini-Mental Status Test (MMST; cutoff < 24; Kessler et al., 1990), the Hospital Anxiety and Depression Scale (HADS; cutoff ≥17; Hinz and Brähler, 2011), and the Patient Health Questionnaire (PHQ; cutoff ≥5; Martin et al., 2006). Eligible participants were scheduled for Visit 2.

Visit 2: Participants completed standardized psychometric tests to assess memory [California Verbal Learning Test (CVLT) (Niemann et al., 2008), Wechsler Memory Scale (WMS) (Härting et al., 2000), and Wechsler Adult Intelligence Scale (WAIS-IV) (Petermann, 2012)], dream characteristics Mannheim Dream Questionnaire [MADRE; (Schredl et al., 2014), sleep quality (Pittsburgh Sleep Quality Index (PSQI) (Hinz et al., 2017)], and mental imagery [Questionnaire of Mental Imagery (QMI) (Sheehan, 1967)].

Polysomnography (PSG): Polysomnography was employed during two consecutive nights in the research sleep laboratory Advanced Sleep Research Berlin to screen for sleep disorders and assess sleep quality. The following parameters were recorded: total sleep time (TST), durations of sleep stages (S1t, S2t, S3t, REMt), sleep onset latency (SL), wake after sleep onset (WASO), and sleep efficiency (SE). In the first sleep night, PSG was additionally used to identify sleep disorders, including obstructive sleep apnea syndrome (OSAS), restless leg syndrome (RLS), and periodic limb movements during sleep (PLMS). During REM sleep, dream activity was assessed through targeted awakenings.

First Sleep Night: Participants were admitted to the Advanced Sleep Research (ASR) facility at 19:30. Following orientation and a re-evaluation of dream activity, PSG equipment was applied between 20:00 and 22:00. Sleep phases were continuously monitored, and participants were awakened twice—once during the third REM phase and once during the fourth REM phase—each after 5 min of stable REM sleep. Dream-recall and -content—if available—was assessed immediately following each awakening by asking “Please excuse the brief interruption to your sleep. Can you remember anything that just happened? Were you dreaming?” Thereafter the dream was protocolled. Morning assessments included a final dream report, verification of sleep parameters, and evaluations of emotional memory using the International Affective Picture System (IAPS; Lang et al., 2008) as well as motor memory using the Finger Tapping Task (FTT; Reitan and Davison, 1974). Participants meeting eligibility criteria were scheduled for the second sleep night. Testing sessions were conducted 24 h apart to ensure consistent intervals between encoding and retrieval phases.

Second Sleep Night: Participants returned to the sleep laboratory for the second session, conducted under identical conditions to the first night, with no REM awakenings to allow for uninterrupted sleep. The morning assessments were conducted in the same manner as after the first night.

### Statistical analysis

For the clinical-descriptive analysis of the collected data and to illustrate the recruitment challenges, the mean values and standard deviations of all metric variables were calculated. Inference statistics were not performed due to small sample size and group differences. Additionally, a confidence interval for the participants' age was determined using exploratory data analysis. Nominal variables were presented using cross-tabulations with corresponding percentage values. The frequencies of the various exclusion reasons were also reported. Statistical analyses were performed using IBM SPSS Statistics, Version 29.0.2.0 (20).

Sleep parameters were extracted manually from standard overnight polysomnography (PSG) reports (REM Logic) for six participants. One participant (ID: 118) was classified as a non-dreamer based on absent dream recall in self-reports and REM awakenings in the sleep lab. The following parameters were selected based on direct report availability and relevance in prior literature (Bischof and Bassetti, 2004):

- REM Sleep (%): Proportion of total sleep time spent in REM sleep.
- Total Arousals: Number of arousals per night; participant 288 was excluded from this metric due to conflicting data.
- Sleep Efficiency (%): Ratio of total sleep time to time in bed.
- REM Latency (min): Time from sleep onset to the first REM episode.

## Results

### Study sample

**Figure 1:**
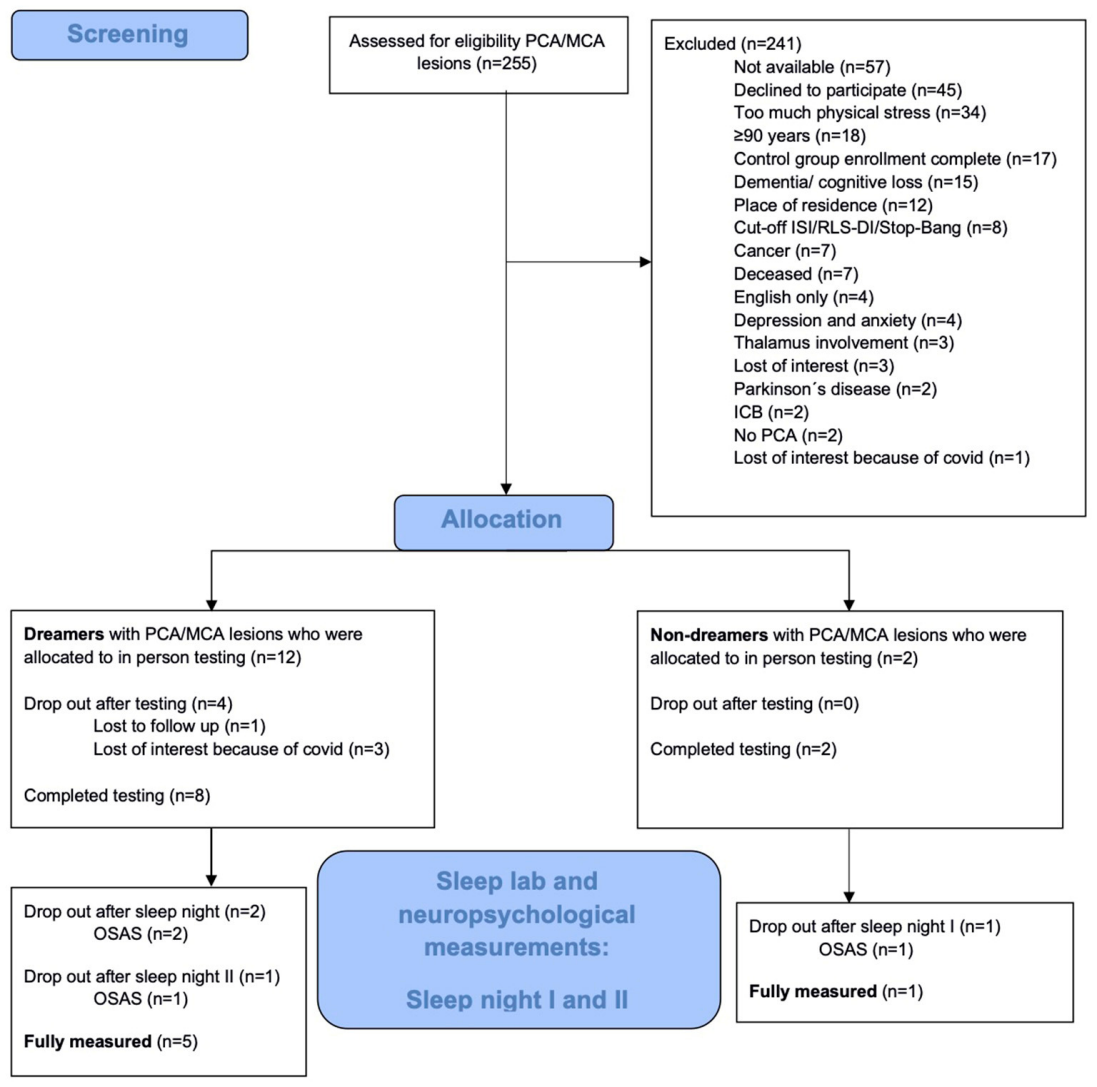
Patient flow diagram.

Between July 2019 and June 2024, a total of 255 patients with PCA ischemic stroke were identified from the Clinic for Neurology at Charité Berlin and the Stroke Research Berlin, consisting of 171 patients from ongoing care on the three university clinic stroke units, and 84 patients via the clinical trial databases 1,000plus and LOBI, as described in the methods section. Due to the nature of the study design and the characteristics of the patient cohort, the screening process was constrained by the limited availability of eligible participants meeting the specific inclusion criteria post-PCA infarction. As a result, the vast majority of individuals had to be classified as screening failures, reflecting both the stringent inclusion and exclusion criteria and the limited pool of eligible patients within the target cohort. The initial 255 patients were assessed for eligibility, but only 14 (5.5%) could ultimately be allocated to the observation groups (Figure 1). The shortage of suitable candidates meant that it was not possible to enroll enough participants to fully populate the experimental group.

The reasons (and number of patients) for recruitment failure were: not meeting the inclusion criteria or meeting exclusion criteria (84), not available via the provided contact information (57), declined participation (45), experiencing too much physical stress (34), insufficient enrollment in experimental group (17), and loss of interest (4).

**Figure 2:**
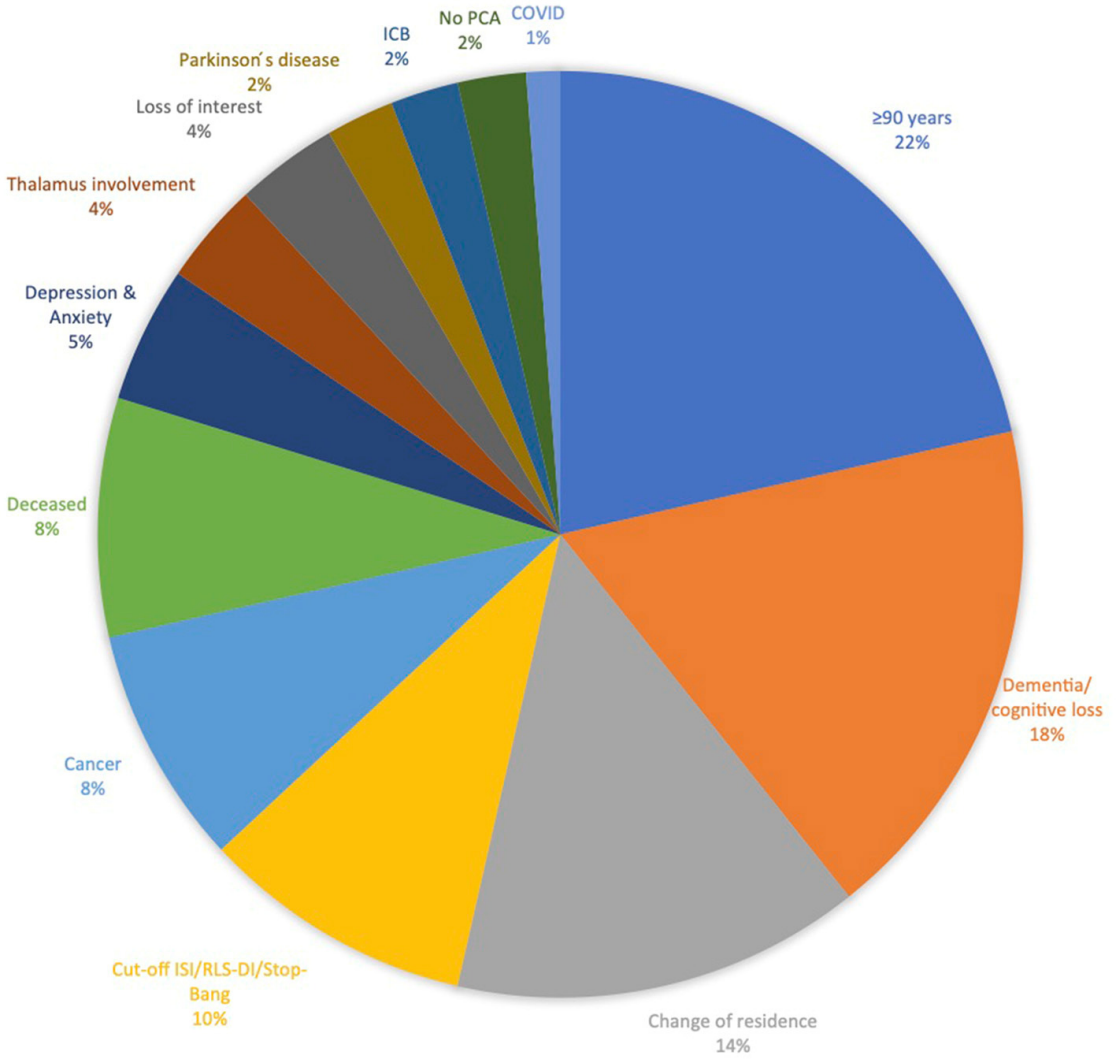
Reasons for exclusion not meeting inclusion or meeting exclusion criteria (*N* = 84).

The most common reason for recruitment failure was that 84 participants did not meet all inclusion criteria or met exclusion criteria, respectively (participants being >90 years old and dementia or cognitive loss were here among the most common reasons; Figure 2).

Of the 14 enrolled participants, 10 subsequently completed the first sleep night in the sleep laboratory. Of the remaining four participants, three dropped out due to loss of interest due to COVID-19 and one was lost to follow-up.

Of the 14 enrolled participants 3 stated in the screening interview in MADRE to be non-dreamers, which we could only verify in 1 participant, by questioning him after REM-sleep in the sleep lab. One dropped out because of obstructive sleep apnea and the other lost interest because of COVID, therefore the dream status of those two participants could not be verified after REM sleep as they were not in the sleep lab. From all 14 included and enrolled participants questionnaire data were elicited regarding Anxiety (HADS-A, PHQ-GAD), Depression (HADS-D, PHQ-DEPR), sleep PSQI and cognitive memory capabilities via CVLT, WMS, WAIS-IV, motor memory by the FTT and dream recall by MADRE. How do those who subjectively report cessation of dream in the screening 1 differ from those who claimed to keep on dreaming? The results given are purely descriptive due to the unequal and small group sizes. We therefore simply report the mean (SD) values for each group. With respect to Anxiety the Non-Dreamers scored on the HADS-A with a mean of 4,00 (SD: 1,73) and on the PHQ-GAD with a mean of 3,34 (4,16) compared to the Dreamers scoring in HADS-A with a mean of 4,30 (SD: 2,95) and on the PHQ-GAD with a mean of 3,00 (SD: 2,79). As for depressiveness results Non-Dreamers showed in HADS-D a mean score of 3,67 (SD: 1,55) vs. HADS-D_Dreamer_ m = 3,00 (SD: 3,23) and PHQ-D_non − Dreamer_ m = 4,34 (SD: 1,53) vs. PHQ-D_Dreamer_ m = 4,27 (SD: 3,41). With respect to insomnia measured by the PSQI the Non-Dreamers scored with a PSQI mean of 6,67 (SD: 4,16) whereas the Dreamers had a PSQI mean of 5,00 (SD: 2,00). In the memory performance tasks the Non-Dreamers performed similar to the Dreamers (WMS, CVLT, WAIS) with the Non-Dreamers having a mean in WMS of 11,33 (SD: 3,79) vs. Dreamers with a WMS mean of 12,11 (SD: 2,37). In CVLT the Non-Dreamers performed with respect to memory consolidation (CVLT DFR II) on average with a mean of 4,67 (SD: 4,51) vs. Dreamers having a mean of 6,10 (SD: 2,60) and with respect to retention rate (CVLT Ret Rate) the Non-Dreamers had a mean of 0,88 vs. Dreamers having a mean of 1,12. As for the Recognition Discrimination (CVLT Rec Discr f p) the Non-Dreamers performed with a mean of 14,00 (SD: 8,66) vs. Dreamers performing with a mean of 8,20 (SD: 1,03). For FTT and IAPS we did not have enough data to describe the two different groups (Table 1).

**Table 1:** Psychological measurements—questionnaires.

**Clinical phenomena**	**Questionnaires**	**Non-dreamer**				**Dreamer**			
		**Mean**	***N***	**SD**	**SE**	**Mean**	***N***	**SD**	**SE**
Anxiety	HADS-A	4.00	3	1.73	1.00	4.30	10	2.95	0.93
	PHQ-GAD	3.34	3	4.16	2.40	3.00	11	2.79	0.84
Depression	HADS-D	3.67	3	1.55	0.67	3.00	10	3.23	1.02
	PHQ-D	4.34	3	1.53	0.88	4.27	11	3.41	1.03
Insomnia	PSQI-TOTAL	6.67	3	4.16	2.40	5.00	11	2.00	0.60
Memory	CVLT LS DG1-4	22.00	3	4.58	2.65	22.60	10	6.72	2.13
	CVLT DFR I	5.33	3	2.52	1.45	5.40	10	3.17	1.00
	CVLT DFR II	4.67	3	4.51	2.60	6.10	10	2.60	0.78
	CVLT Ret Rate	0.88	3			1.12	10		
	CVLT Rec Discr f p	14.00	3	8.66	5.00	8.20	10	1.03	0.33
	WMS	11.33	3	3.79	2.19	12.11	9	2.37	0.79
	WAIS-IV sim	10.00	3	3.00	1.73	10.80	10	3.26	1.03
	WAIS-IV nbrs	7.00	3	5.19	3.00	9.90	10	1.19	0.38
	WAIS-IV voc	12.67	3	1.15	0.67	10.50	10	2.72	0.86

Descriptive results. HADS-A/HADS-D, Hospital Anxiety and Depression Scale clinical cutoff ≥17; PHQ, Patient Health Questionnaire (PHQ-GAD, for General Anxiety Disorder; PHQ-D, for Depression) clinical cutoff ≥5; PSQI, Pittsburgh Sleep Quality Index (0–4, good sleep quality, 5–10 moderate sleep problems, ≥10 significant sleep problems); CVLT, California Verbal Learning Test (DFR, delayed free recall [memory consolidation], Ret Rate, retention rate [DFR II ÷ DFR I], Rec Discr f p, Recognition Discrimination false positive); WMS, Wechsler Memory Scale; WAIS-IV, Wechsler Adult Intelligence Scale.

After the first sleep night, 3 participants were excluded due to the diagnosis of Obstructive Sleep Apnea Syndrome (OSAS). After the second sleep night, one additional participant was excluded due to OSAS.

**Figure 3:**
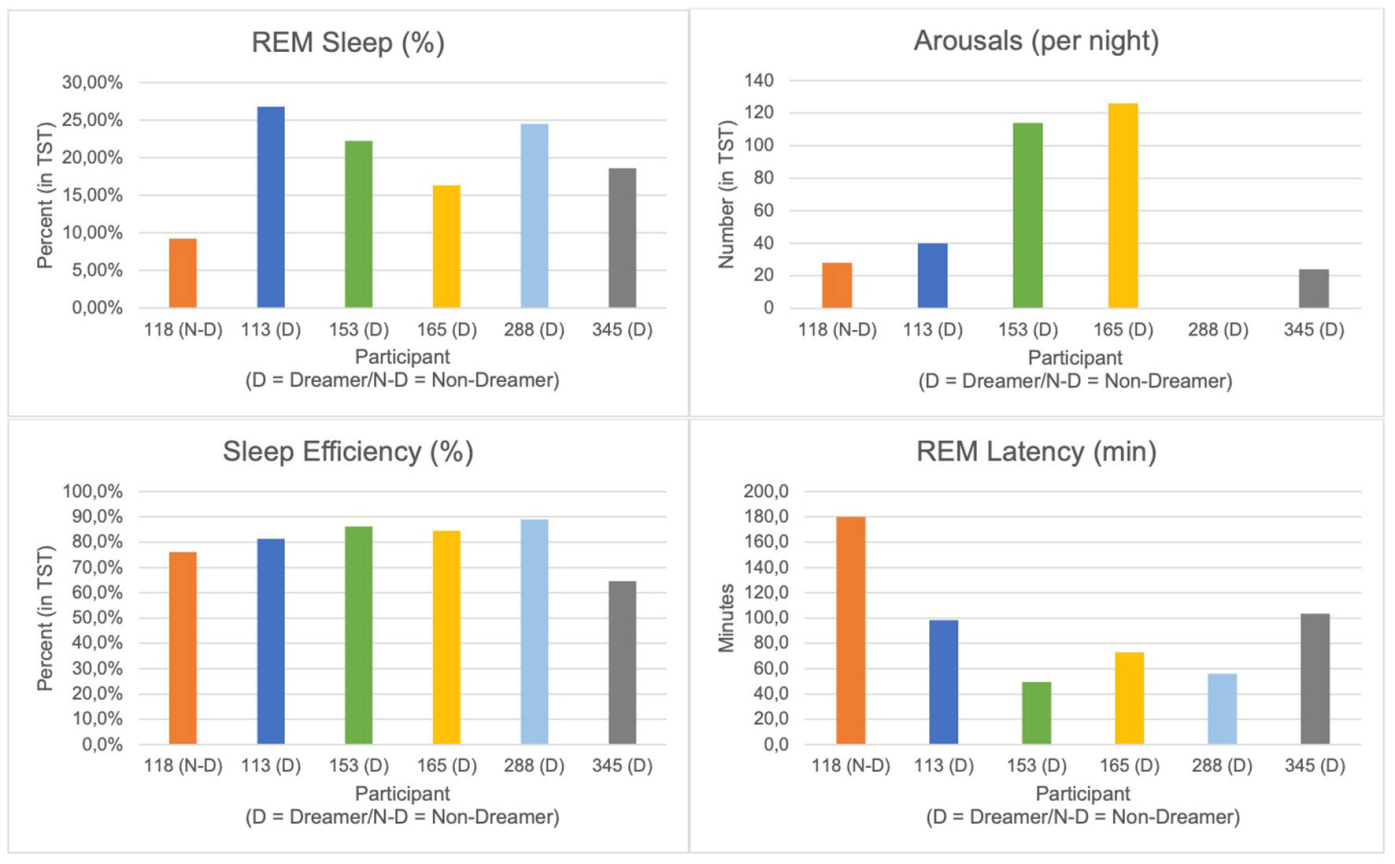
PSG data.

The remaining 6 participants (*N* = 5 Dreamers; *N* = 1 Non-Dreamer) who successfully completed the first sleep night demonstrated the following sleep parameters (see Table 2 and Figure 3). The Non-Dreamer (118) showed the lowest REM sleep percentage (9.2%) and a long REM latency (106 min). While the Non-Dreamer's total arousal count (28) was lowest, their overall sleep efficiency (86.2%) was comparable to those of the Dreamers. In general, participants with better dream recall tended to show shorter REM latency and higher REM proportions (apart from one exception: participant 345), more arousals (per night) and better sleep efficiency.

**Table 2:** Valid sleep parameters dreamers vs. non-dreamer.

**Participant**	**REM sleep [%]**	**Arousals (per night)**	**Sleep efficiency [%]**	**REM latency [min]**
118 (Non-dreamer)	9.2	28	86.2	106.0
113	26.8	40	89.5	87.5
153	22.2	114	83.3	63.5
165	16.3	126	71.9	92.0
288	24.5	(no valid data)	89.0	103.5
345	18.6	70	64.6	103.5

### Comparison of pilot completers and all screened participants

To understand the reasons for the imbalance between numbers of non-completers (*N* = 249) and completers (*N* = 6), we analyzed clinical characteristics of the two groups to identify potential factors contributing to the high number of screening failures.

### Age and gender distribution

**Figure 4:**
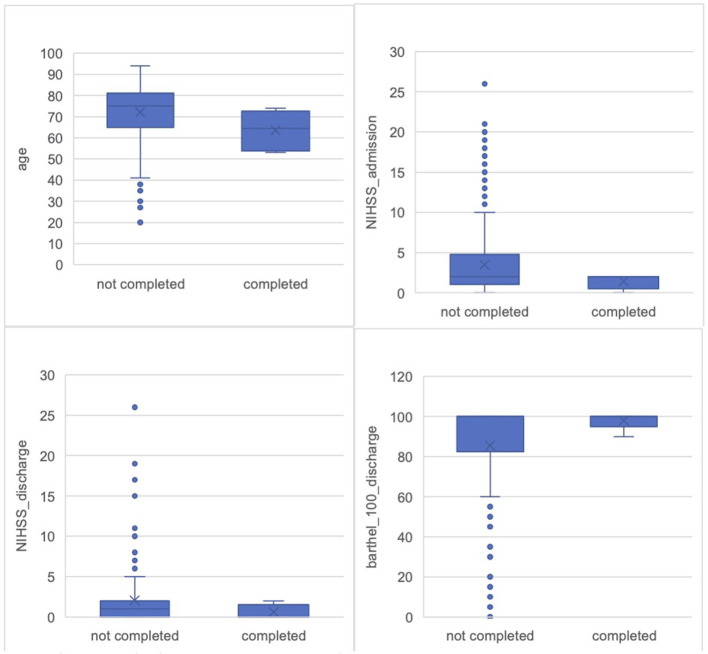
Comparison of all screened participants and pilot completers. Age: Non-completers M = 72.13 years (SD = 12.92, SE = 0.82); Completers M = 63.67 years (SD = 9.22; SE = 3.77). NIHSS_admission: Non-completers M = 3.47 (SD = 4.6, SE = 0.33); Completers M = 1.40 (SD = 0.89; SE = 0.40). NIHSS_discharge: Non-completers M = 2.03 (SD = 3.71, SE = 0.27); Completers M = 0.60 (SD = 0.89; SE = 0.40). Bartel_index: Non-completers M = 85.54 (SD = 25.87, SE = 2.1); Completers M = 98.00 (SD = 4.47; SE = 2.0).

The average age in the completion group was 63.67 years (SD = 9.22; 95% CI = 53.99–73.35). In the non-completers group, the average age was 73.13 years (SD = 12.92; 95% CI = 70.52–73.74) (Figure 4). The gender distribution across both groups showed a predominance of male participants. In the non-completion group, 155 participants were male (62.2%) and 94 participants were female (37.8%). In the completion group, 5 participants were male (83.3%) and 1 participant was female (16.7%). Overall, gender analysis showed that a male majority was consistent across both groups.

### Initial stroke severity (NIHSS scores)

Participants in the completion group showed a lower initial stroke severity, as indicated by the NIHSS (National Institutes of Health Stroke Scale) scores (Brott et al., 1989), a standardized tool to objectively quantify the impairment caused by a stroke. Scores range from 0 to 42, with higher scores indicating greater stroke severity and worse neurological deficits (Brott et al., 1989). The average NIHSS score at admission for the completion group (data for *N* = 5^2^) was 1.40 (SD = 0.89), while it was higher in the non-completion group (data for *N* = 200^3^), with an average of 3.47 (SD = 4.60) (Figure 4). Upon discharge, the NIHSS score in the completion group was further reduced, averaging 0.60 (SD = 0.89), compared to 2.03 (SD = 3.71) in the non-completion group (Figure 4). Greater stroke severity, as reflected by higher NIHSS scores, was more common in the non-completion group.

### Lysis and recanalization procedures

Lysis and recanalization procedures are essential interventions following acute stroke admission. These interventions are critical in minimizing brain damage and improving patient outcomes when administered promptly (He et al., 2024). In the completion group [data for *N* = 5 (see footnote 1)], 1 participant (16.1%) did undergo lysis, while in the non-completion group (data for *N* = 210^4^) 41 participants (16.5%) did undergo lysis. Recanalization was performed in 1 participant (16.7%) of the completion group [data for *N* = 5 (see footnote 1)] and 26 participants (10.4%) of the non-completion group (data for *N* = 198^5^).

### Functional outcomes and everyday deficits

Participants who completed the study demonstrated better functional outcomes, as reflected in both discharge Barthel Index scores and everyday deficits documented in the medical reports. The Barthel Index, a widely recognized measure of functional independence, evaluates a patient's ability to perform basic activities of daily living. Scores range from 0 to 100, with higher scores representing greater independence (Mahoney and Barthel, 1965). In the completion group (data for *N* = 5^6^) the average Barthel score was 98.00 (SD = 4.47), indicating a high level of independence. In contrast, the non-completion group (data for *N* = 157^7^) had an average discharge Barthel score of 85.54 (SD = 25.87) (Figure 4).

Everyday deficits analysis further supported these findings. In the completion group (*N* = 6), 4 participants (66.7%) had no functional impairments, while 2 participants (33.3%) exhibited light, defined as one functional impairment, with none presenting medium or severe impairments, defined as three or more deficits. Conversely, in the non-completion group (data for *N* = 207^8^), 76 participants (30.5%) had no functional impairments, 71 participants (28.5%) had light functional impairments, 28 participants (11.2%) had medium impairments, and 32 participants (12.9%) had severe impairments.

### Discharge destinations

The discharge destinations differed between the two groups, reflecting the pathways participants followed after their hospital stay. In the completion group (*N* = 6), the majority of 5 participants (83.3%), were discharged home, and 1 participant (16.7%) was discharged home with a rehabilitation arranged. In the non-completion group (data for *N* = 211^9^), 130 participants (52.2%) were discharged home, followed by 33 participants (13.3%) discharged to rehabilitation facilities, 17 participants (6.8%) to nursing homes, and 15 participants (6.0%) being discharged home with rehabilitation arranged. Additionally, 7 participants (2.8%) left against medical advice, 5 participants (2.0%) were transferred to another ward, 3 participants (1.2%) died, and 1 participant (0.4%) was discharged as homeless.

## Discussion

This methodological report aims to evaluate whether patients with an ischemic posterior cerebral artery (PCA) infarction who lose the ability to recall dreams demonstrate alterations in sleep quality and memory consolidation, compared to those retaining dream recall. Although anchored in a robust theoretical framework, the report reveals several methodological challenges, notably in recruiting a clinical cohort with such restrictive eligibility criteria. Of 255 screened PCA patients, only 14 were eligible, with just 6 ultimately completing both polysomnography (PSG) assessments and final testing. This is largely attributed to the rarity of isolated ischemic infarcts in the PCA region without adjacent brainstem or thalamic involvement, which significantly narrowed the patient pool (Rennert et al., 2019).

The recruitment difficulties, echoing prior neurological research challenges (Berge et al., 2016; Broderick et al., 2023), highlight the issue of attrition, where “Completers” differed significantly from “Non-Completers.” The Completers were generally younger, had lower NIHSS scores at admission, and were nearly fully independent on the Barthel Index at discharge. These observations are consistent with previous findings where stroke severity and older age hindered participation in complex research protocols (Kwakkel et al., 1996). Most Completers' discharge to home supports their better clinical status, aligning with literature that highlights more severe deficits often lead to discharge to rehabilitation or nursing facilities (Alexander, 1994). The low prevalence of isolated ischemic infarcts in the PCA territory without brainstem or thalamic involvement significantly reduced recruitment rates. While PCA infarcts are already less common than anterior-circulation strokes, restricting enrollment to purely parieto-occipital lesions (excluding brainstem and thalamus) further narrowed the patient pool (Rennert et al., 2019). Consequently, achieving an adequate sample size becomes challenging even when recruitment is conducted in major university clinic stroke centers.

The high attrition rate in this trial resonates with prior research of neurological populations (Berge et al., 2016; Broderick et al., 2023). A particularly noteworthy finding pertains to the stark differences between those who completed the study (“Completers”) and those who dropped out or were excluded during the screening or data collection phase (“Non-Completers”). Completers were on average younger, had distinctly lower NIHSS scores upon admission and showed almost complete functional independence in the Barthel Index at discharge.

These findings align well with previous stroke research where lower NIHSS and older age often making participation in complex research protocols more difficult (Kwakkel et al., 1996).

The fact that most Completers could be discharged home suggests a comparatively better overall clinical condition, which is consistent with the literature describing that more severe deficits (e.g., aphasia, significant motor impairment) often result in discharge to rehabilitation or nursing facilities (Alexander, 1994). Within the present study, individuals with milder stroke courses were more likely to see the protocol through.

No substantial differences in the use of thrombolysis and recanalization were observed between Completers and Non-Completers, suggesting lesion severity rather than acute treatment primarily influenced study participation. Additionally, the emergence of sleep disorders like obstructive sleep apnea (OSA) during PSG nights disqualified further participants. This concern is well-documented, noting OSA's prevalence in post-stroke populations (Khot and Morgenstern, 2019). Future methodologies might benefit from incorporating improved pre-screening procedures for OSA to refine participant selection.

The following discussion of PSG-derived sleep parameters is deliberately brief, as the data are purely descriptive and the small sample size precludes any meaningful inferential statistical conclusions. Nevertheless, we observed that the sleep architecture of our single Non-Dreamer, including REM duration, latency and density, remained intact, while dreaming ceased, similar to what was reported by Bischof and Bassetti (2004) in a case of complete dream loss following bilateral occipital stroke (Charcot-Wilbrand syndrome). So, to a certain degree this supports the notion that dream generation and REM sleep physiology can be decoupled. However, it should be noted that our Non-Dreamer exhibited among the lowest percentage of REM sleep, the longest REM latency, while maintaining comparably good sleep efficiency and a total arousal count that was surprisingly low. These results necessitate validation through larger-scale studies to explore the underlying mechanisms in dream loss further (Solms, 2000).

The selection of PCA stroke as the focus was premised on evidence that occipital and parieto-occipital lesions may terminate dreaming while preserving REM sleep, presenting a nuanced opportunity to examine the effects of dream content cessation on sleep continuity and memory processing (Bischof and Bassetti, 2004). Yet, the recruitment challenges encountered suggest that PCA stroke, in reality, may not be the most effective lesion model for expansive examination of dream function.

Two primary recommendations for future research emerge from this methodology. Firstly, the observed dropout rate and recruitment challenges question the practicality of disentangling dreams' function from REM sleep strictly through lesion-based methods. Despite the conceptual appeal of analyzing the potential role of dreams in sleep maintenance and memory consolidation, the current data indicate that pursuing such inquiries may require either larger recruitment networks or less stringent criteria.

Secondly, given the complexities inherent in executing specialized protocols within vulnerable and heterogenous populations, employing “functional lesion” models in healthy individuals could be an innovative future direction. Techniques like transcranial alternating current stimulation (tACS) or transcranial magnetic stimulation (TMS) could transiently modulate parieto-occipital areas during REM or pre-sleep states, thus temporarily affecting dream recall in a controlled environment (Blanchette-Carrière et al., 2020; Noreika et al., 2020; Scarpelli et al., 2021; Voss et al., 2014). Such methods would allow repeated measures within subjects, mitigating many confounds present in stroke populations.

## Data Availability

The raw data supporting the conclusions of this article will be made available by the authors, without undue reservation.

## Footnotes

^1^Dreaming refers here and throughout the article to dream recall.

^2^*N*= 1 missing data for NIHSS, Lysis and Recanalization.

^3^*N*= 49 missing data for NIHSS.

^4^*N*= 39 missing data for lysis.

^5^*N*= 51 missing data for Recanalization.

^6^*N*= 1 missing data for Barthel score.

^7^*N*= 92 missing data for Barthel score.

^8^*N*= 42 missing data for Everyday deficits.

^9^*N*= 38 missing data for discharge destination.
